# Fedratinib Attenuates Bleomycin-Induced Pulmonary Fibrosis via the JAK2/STAT3 and TGF-β1 Signaling Pathway

**DOI:** 10.3390/molecules26154491

**Published:** 2021-07-26

**Authors:** Hao Ruan, Jiaoyan Luan, Shaoyan Gao, Shuangling Li, Qiuyan Jiang, Rui Liu, Qing Liang, Ruiqin Zhang, Fangxia Zhang, Xiaohe Li, Honggang Zhou, Cheng Yang

**Affiliations:** 1 State Key Laboratory of Medicinal Chemical Biology, College of Pharmacy and Key Laboratory of Molecular Drug Research, Nankai University, Tianjin 300000, China; ruanhao8958@mail.nankai.edu.cn (H.R.); 13553118700@163.com (J.L.); ShaoyanGao339@163.com (S.G.); 2120191206@mail.nankai.edu.cn (S.L.); Tiancha18822188139@163.com (Q.J.); lauraiz@163.com (R.L.); 15802252982@163.com (Q.L.); zhangruiqin1996@126.com (R.Z.); fangxiazhangcc@163.com (F.Z.); lixiaohe908@163.com (X.L.); cheng.yang@nankai.edu.cn (C.Y.); 2 High-Throughput Molecular Drug Screening Centre, Tianjin International Joint Academy of Biomedicine, Tianjin 300070, China

**Keywords:** fedratinib, pulmonary fibrosis, JAK2/STAT3, transforming growth factor-β1, fibroblast-to-myofibroblast transition

## Abstract

Idiopathic pulmonary fibrosis (IPF) is a chronic, progressive interstitial lung disease with multiple causes, characterized by excessive myofibrocyte aggregation and extracellular matrix deposition. Related studies have shown that transforming growth factor-β1 (TGF-β1) is a key cytokine causing fibrosis, promoting abnormal epithelial–mesenchymal communication and fibroblast-to-myofibroblast transition. Fedratinib (Fed) is a marketed drug for the treatment of primary and secondary myelofibrosis, targeting selective JAK2 tyrosine kinase inhibitors. However, its role in pulmonary fibrosis remains unclear. In this study, we investigated the potential effects and mechanisms of Fed on pulmonary fibrosis in vitro and in vivo. In vitro studies have shown that Fed attenuates TGF-β1- and IL-6-induced myofibroblast activation and inflammatory response by regulating the JAK2/STAT3 signaling pathway. In vivo studies have shown that Fed can reduce bleomycin-induced inflammation and collagen deposition and improve lung function. In conclusion, Fed inhibited inflammation and fibrosis processes induced by TGF-β1 and IL-6 by targeting the JAK2 receptor.

## 1. Introduction

Idiopathic pulmonary fibrosis (IPF) is a chronic, progressive interstitial lung disease with a poor prognosis with unknown etiology. IPF is characterized by damage in the epithelium and fibrogenesis in the interstitial tissue, leading to progressing alveolar structure destruction and pulmonary function failure. Patients diagnosed with PF have a median survival of only 2−4 years [1]. Pharmacotherapy for PF is emerging in recent years. The availability of nintedanib and pirfenidone provides patients with more flexible therapeutic options. However, clinical outcomes reveal them to simply mitigate symptoms or retard progression and to fail to prolong survival. Given this situation, more pharmacotherapy strategies are needed for patients to improve their health status.

Alveolar epithelial cell (AEC) dysfunction following epithelial injury initiates fibrosis. Injured AECs secret numerous mediators that induce the migration of mesenchymal cells of different origins (such as local fibroblasts or fibrocytes), as well as their differentiation to myofibroblasts [2]. These myofibroblasts in turn contribute to excessive extracellular matrix (ECM) deposition and eventually destroy the lung architecture. Some epithelial cells are also reprogrammed to overexpress mesenchymal markers that contributes to the breakdown of the epithelial junction and lung interstitial remodeling. Transforming growth factor β1 (TGF-β1), tumor necrosis factor α (TNFα) and interleukin (IL)-1β are all expressed by AECs and promote profibrotic responses. Among all these factors, TGF-β1 is the most potent profibrotic mediator. Exaggerated TGF-β1 promotes aberrant wound healing of AECs and fibroblast-to-myofibroblast transition (FMT) through multiple signaling pathways [3]. Although TGF-β1 is an attractive target for IPF therapy, few candidates have reached late-phase clinical trials due to their toxicity, e.g., BG00011, a humanized monoclonal antibody directed against integrin αvβ6 (NCT01371305). These factors drive us to explore molecules targeting a less toxic pathway that exhibits crosstalk with TGF-β1 signaling.

Janus kinase (JAK) is a receptor-related tyrosine kinase that plays a central role in multiple homeostatic processes including inflammation responses [4], apoptosis [5] and cell proliferation [6]. JAK2 initiates responses to several inflammatory factors such as IL-6 [7] and interferon (IFN)-γ [8], and JAK2/STAT3 is also a non-classical signaling pathway of TGF-β1 [9]. Once activated, JAK kinase recruits and phosphorylates signal transducer and activator of transcription (STAT) proteins, following STATs’ dimerization and translocating into the nucleus, where they activate the transcription of several target genes. Alterations in JAK2 signaling cause profound responses. STAT3 phosphorylation participates in both lung epithelial cell damage and FMT [10,11] and contributes to fibrogenesis [12]. Studies also revealed that JAK2/STAT3 signaling undergoes a hyperactivation in IPF patients [12]. Therefore, targeting JAK2/STAT3 signaling has been highlighted as a potential strategy for treating pulmonary fibrosis.

Fed is a JAK2-selective kinase inhibitor recently approved by the U.S. Food and Drug Administration (FDA) for the treatment of myofibrosis (MF) [13]. Clinical study shows that patients with MF receiving Fed have benefited from spleen-size reductions and have a significantly lower level of several inflammatory factors such as tumor necrosis factor α (TNF-α) [14]. In this study, we demonstrate that Fed can inhibit the progression of pulmonary inflammation and fibrosis by inhibiting both JAK2/STAT3 and TGF-β1 signaling. In vitro study shows that Fed inhibits TGF-β1- and IL-6-induced hyperactivity of epithelial cells and FMT and reduces inflammatory factor expression of epithelial cells. Further study confirms its in vitro antifibrotic effect, manifesting as the decrease of fibroblast migration and activation and downregulation of TGF-β1/Smad and non-Smad signaling. In vivo studies reveal the same result. These findings together demonstrate the antifibrotic effects of Fed and might have direct translational implications.

## 2. Results

### 2.1. Fedratinib Inhibited Mesenchymal Marker Expression in Epithelial Cells and Fibroblast-to-Myofibroblast Transition by Suppressing JAK2/STAT3 Signaling

**Figure 1:**
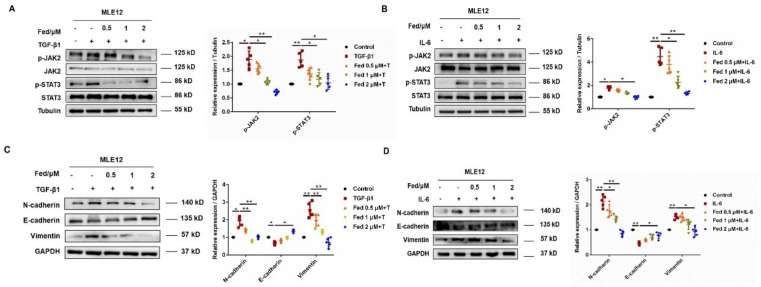
Fedratinib inhibits mesenchymal marker expression in epithelial cells through inhibiting JAK2/STAT3 signaling. (**A**,**B**) Ratios of JAK2/p-JAK2 and STAT3/p-STAT3 were determined by Western blotting in MLE12 cells stimulated for 24 h with TGF-β1 (5 ng/mL) or IL-6 (50 ng/mL) at different concentrations of fedratinib. (**C**,**D**) MLE12 cells were treated with TGF-β1 or IL-6 and Fed (0.5, 1 and 2 μM) for 24 h. Protein expression levels of E-cadherin, vimentin and N-cadherin were assessed by Western blot. GAPDH was used as a loading control in grayscale analysis. Data are presented as the means ± SD (*n* = 5). Comparisons between groups were achieved using one-way analysis of variance (ANOVA). * *p* < 0.05, ** *p* < 0.01.

**Figure 2:**
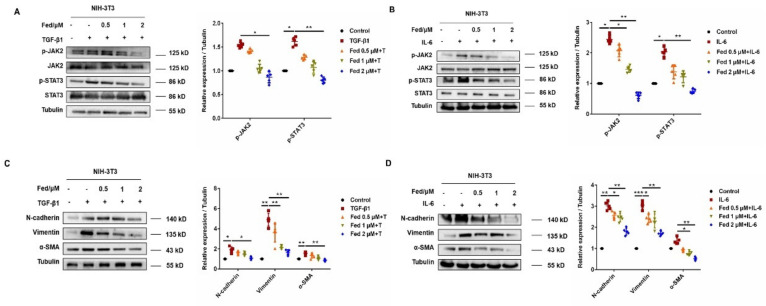
Fedratinib inhibits fibroblast-to-myofibroblast transitions by inhibiting the JAK2 and STAT3 signaling pathways. (**A**,**B**) JAK2/p-JAK2 and STAT3/p-STAT3 protein expression in NIH-3T3 cells. (**C**,**D**) NIH-3T3 cells were treated with TGF-β1 or IL-6 and Fed (0.5, 1 and 2 μM) for 24 h. Protein expression levels of E-cadherin, vimentin and N-cadherin were assessed by Western blot. β-tubulin was used as a loading control in grayscale analysis. Data are presented as the means ± SD (*n* = 5). Comparisons between groups were achieved using one-way analysis of variance (ANOVA). * *p* < 0.05, ** *p* < 0.01, *** *p* < 0.001.

We first established an AEC injury model by stimulating MLE12 with TGF-β1. TGF-β1 significantly enhanced JAK2 and STAT3 phosphorylation levels but downregulated by Fed in a dose-dependent manner (Figure 1A). TGF-β1 stimulation also promoted MLE12 to increasingly express mesenchymal markers, N-cadherin and vimentin, and decreasing the epithelial marker, E-cadherin, while Fed attenuated these changes (Figure 1C). Since IL-6 is a crucial inflammatory factor initiating JAK2/STAT3 signaling and playing an important role in epithelial senescence [15] and wound repair [16], we also stimulated MLE12 with IL-6 and investigated the efficacy of Fed. IL-6 also increased JAK2 and STAT3 phosphorylation and mesenchymal marker expression, while these phenotypes were reversed by Fed administration (Figure 1B,D). Similar results were observed in lung fibroblasts. We established an FMT model by stimulating NIH-3T3 with TGF-β1 or IL-6. The phosphorylation levels of JAK2 and STAT3 were significantly increased in both models and were suppressed by Fed, not surprisingly (Figure 2A,B). Stimulation of NIH-3T3 with TGF-β1 or IL-6 increased the expression of both myofibroblast marker, α smooth muscle actin (α-SMA), and mesenchymal markers, N-cadherin and vimentin, while Fed reversed these changes in a dose-dependent manner (Figure 2C,D). These data together reveal that Fed is capable of inhibiting mesenchymal marker expression in epithelial cells and fibroblast-to-myofibroblast transition through inhibiting JAK2/STAT3 signaling.

### 2.2. Fed Regulated the Production of Inflammatory Cytokines in Lung Epithelial Cells

**Figure 3:**
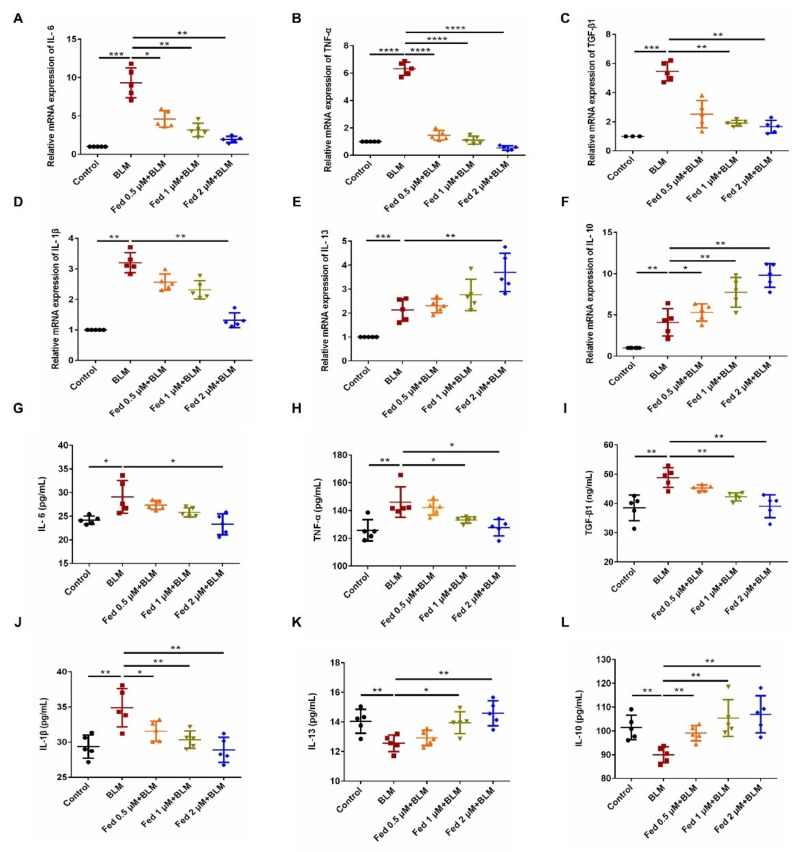
Fedratinib regulated the production of inflammatory cytokines in lung epithelial cells. (**A**–**F**) MLE12 cells were treated with BLM (5 U) and Fed (0.5, 1 and 2 μM) for 24 h. TGF-β1, TNF-α, IL-1β, IL-6, IL-13 and IL-10 were analyzed by real-time PCR in MLE12 cells. (**G**–**L**) MLE12 cells were treated with BLM (5 U) and Fed (0.5, 1 and 2 μM) for 24 h. The expression of inflammatory factors including TGF-β1, TNF-α, IL-1β, IL-6, IL-13 and IL-10 in cell supernatant was detected using an ELISA kit. Data are presented as the means ± SD (*n* = 5). Comparisons between groups were achieved using one-way analysis of variance (ANOVA). * *p* < 0.05, ** *p* < 0.01, *** *p* < 0.001, **** *p* < 0.0001.

Injured epithelial cells produce numerous cytokines and promote fibrogenesis. Thus, we further analyzed the effect of Fed on cytokine production in alveolar epithelial cells. Bleomycin (BLM) is a glycopeptide antibiotic that exhibits potent anticancer activities [17] and is recognized to induce epithelial cell injury after exposing to lung [18]. Thus, we stimulated MLE12 cells with BLM to construct an epithelial cell injury model and harvested 24 h after Fed incubation. The qPCR results showed that Fed administration significantly decreased the expression of profibrotic cytokines, TGF-β1, TNF-α, IL-1β and IL-6, while it elevated the expression of antifibrotic factors IL-10 and IL-13 (Figure 3A–F). We also harvested the cell supernatant to measure the secretion levels of these cytokines by ELISA, and results showed that profibrotic cytokines IL-6, TNF-α and TGF-β1 and IL-1β were decreased by Fed treatment, while the antifibrotic factors IL-10 and IL-13 were elevated (Figure 3G–L). Notably, although the mRNA levels of IL-10 and IL-13 were increased after BLM stimulation, the secretion levels were decreased. These results indicate that Fed can regulate the cytokine production of lung epithelial cells. Since profibrotic mediators such as IL-6 and TGF-β1 will in turn enhance the epithelial cell injury, Fed might prevent such a vicious circle.

### 2.3. Fed Suppresses TGF-β1-Induced Myofibroblasts Proliferation, Migration, Activation and Apoptosis Resistance

**Figure 4:**
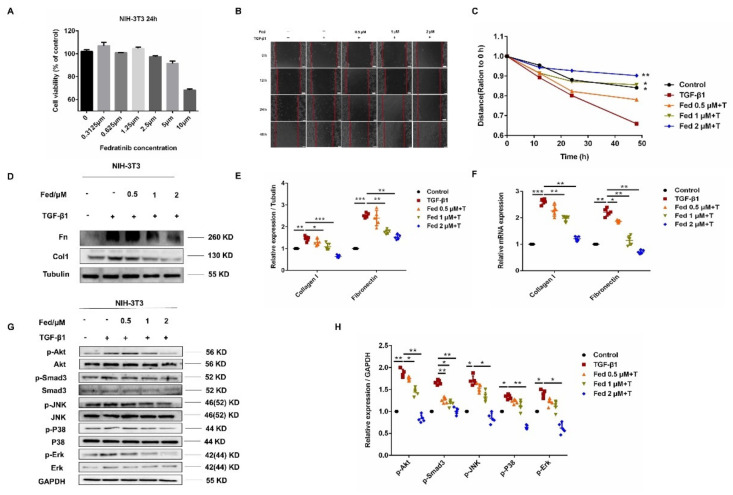
Fedratinib suppresses TGF-β1-induced myofibroblast proliferation, migration and activation. (**A**) MTT assays of NIH-3T3 cells. Cells were exposed to the indicated doses of Fed (0 to 10 μM) for 24 h, IC_50_ = 11 μM. (**B**,**C**) A wound healing assay was used to assess the effect of Fed on fibroblast migration. The wound closure was photographed at 0, 12, 24 and 48 h post-scratching. Experiments were performed in triplicate. (**D**,**E**) NIH-3T3 cells were treated with TGF-β1 (5 ng/mL) and Fed (0.5, 1 and 2 μM) for 24 h. The protein levels of Col1 and Fn were analyzed by Western blot. (**F**) The mRNA levels of Col1 and Fn were analyzed by real-time PCR. (**G**,**H**) The phosphorylation levels of Smad3, P38, JNK, Erk and Akt were analyzed by Western blot in NIH-3T3. β-tubulin and GAPDH were used as loading control in grayscale analysis. Data are presented as the means ± SD (*n* = 5). Comparisons between groups were achieved using one-way analysis of variance (ANOVA). * *p* < 0.05, ** *p* < 0.01, *** *p* < 0.001.

**Figure 5:**
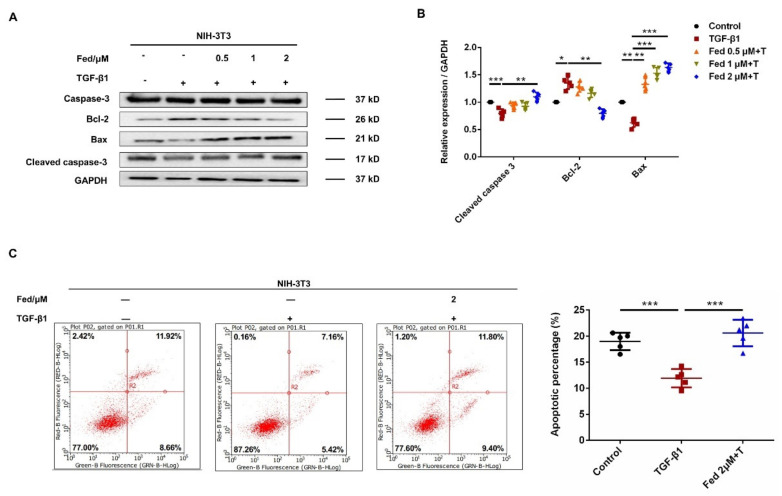
Fedratinib suppresses TGF-β1-induced myofibroblast apoptosis resistance. (**A**,**B**) NIH-3T3 cells were treated with TGF-β1 (5 ng/mL) and Fed (0.5, 1 and 2 μM) for 24 h. The protein levels of caspase-3, Bcl-2 and Bax were analyzed by Western blot. GAPDH was used as a loading control in grayscale analysis. (**C**) NIH-3T3 cells were treated with TGF-β1 (5 ng/mL) and Fed (2 μM) for 24 h. The percentage of apoptosis was determined by flow cytometry. Data are presented as the means ± SD (*n* = 5). Comparisons between groups were achieved using one-way analysis of variance (ANOVA). * *p* < 0.05, ** *p* < 0.01, *** *p* < 0.001.

To detect whether Fed has an influence on the proliferation of TGF-β1-induced fibroblasts, NIH-3T3 cells were treated with or without TGF-β1 (5 ng/mL) and different doses of Fed for 24 h. We found that the IC_50_ value of Fed in the NIH-3T3 cells was 11 μM (Figure 4A). We further evaluated the in vitro antifibrosis effect of Fed using a classical TGF-β1-induced model. NIH-3T3 cells stimulated with TGF-β1 showed an accelerated migration, but this was inhibited by Fed in a dose-dependent manner, and the level of fibrosis in the high-dose group (2 μM) was almost equal to that of the control group (Figure 4B,C). We then tested the effect of Fed on TGF-β1-induced myofibroblast hyperactivation. Results showed that Fed decreased both the protein and mRNA level of key profibrotic factors, collagen I (Col1) and fibronectin (Fn), that had been enhanced by TGF-β1, indicating its potential effects in reducing ECM deposition (Figure 4D–F). Further study also revealed that Fed could suppress TGF-β1-induced Smad and non-Smad activation, manifesting as the downregulation of p-Smad3, p-Akt, p-Erk, p-P38 and p-JNK levels (Figure 4G,H). This result is not surprising since JAK2/STAT3 has crosstalk with TGF-β1 signaling, indicating that Fed displays antifibrotic effects through targeting multiple kinase signaling. Finally, myofibroblasts undergo an apoptosis-resistance phenotype and contribute to fibrotic foci formation and ECM deposition. We also studied the effect of Fed on apoptosis. TGF-β1-induced NIH-3T3 exerted an apoptosis-resistance phenotype as the expression of anti-apoptotic protein Bcl-2 showed an increase and pro-apoptotic Bax and cleaved caspase-3 showed a decrease (Figure 5A,B). This result was confirmed by the flow cytometry assay. TGF-β1 stimulation reduced the percentage of apoptotic cells to 12.58%, while Fed administration remarkably increased the percentage to 21.2% (Figure 5C). These data together demonstrate that, remarkably, Fed can suppress TGF-β1-induced myofibroblasts migration, activation and apoptosis resistance by inhibiting not only JAK2/STAT3 signaling but also TGF-β1/Smad and non-Smad.

### 2.4. Influence of Fed on Bleomycin-Induced Lung Fibrosis and Inflammation

**Figure 6:**
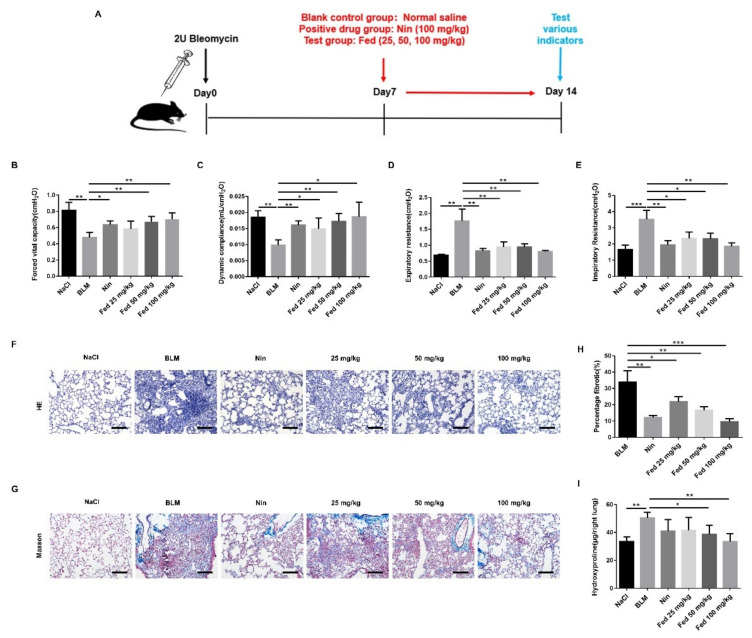
Influence of fedratinib on bleomycin-induced lung fibrosis. (**A**) Dosing regimen in BLM-induced pulmonary fibrosis model. (**B**) Forced vital capacity (FVC). (**C**) Dynamic compliance (Cdyn). (**D**) Expiratory resistance (Re). (**E**) Inspiratory resistance (Ri). (**F**) Lung tissue sections were stained with hematoxylin and eosin staining (H&E). (**G**) Lung tissue sections were stained with Masson trichrome staining. (**H**) Statistics of lung fibrosis area among groups. (**I**) Hydroxyproline (HYP) contents of lung tissues in mice. Scale bar = 50 μm. Data are presented as the means ± SD (*n* = 8). Comparisons between groups were achieved using one-way analysis of variance (ANOVA). * *p* < 0.05, ** *p* < 0.01, *** *p* < 0.001.

To investigate the influence of Fed on pulmonary fibrosis, we challenged C57BL/6 mice with BLM. Previous studies have suggested that bleomycin-induced oxidative stress and inflammatory response contribute to the development of pulmonary fibrosis [19,20]. Although inadequacies still exist, BLM-induced pulmonary fibrosis has been described as “the best-characterized animal model available for preclinical testing” of IPF. Different doses of Fed were gavaged from day 7 to day 14 after surgery, and nintedanib was used as a positive control (Figure 6A). As shown in Figure 6, BLM treatment resulted in distinct fibrosis foci formation and alveolar structure destruction, while H&E staining and quantification of fibrotic lung sections illustrated that Fed attenuated fibrogenesis in a dose-dependent manner (Fed 100 mg/kg vs. BLM, *p* = 0.0006, Figure 6F,H). Collagen content was significantly decreased in mice treated with Fed. The high-dose group had a remarkably less collagen content compared with the BLM group (Fed 100 mg/kg vs. BLM, *p* = 0.0013, Figure 6I) and was better than Masson staining, revealing a similar trend (Figure 6G). We also measured the alteration of pulmonary functions in mice. The forced vital capacity (FVC) and dynamic compliance (CyDn) were shown to be decreased after BLM challenge, while inspiratory resistance (Ri) and expiratory resistance (Re) were increased. Fed treatment significantly reversed these changes (Figure 6B–E). These results demonstrate the therapeutic effects of Fed on BLM-induced pulmonary fibrosis.

**Figure 7:**
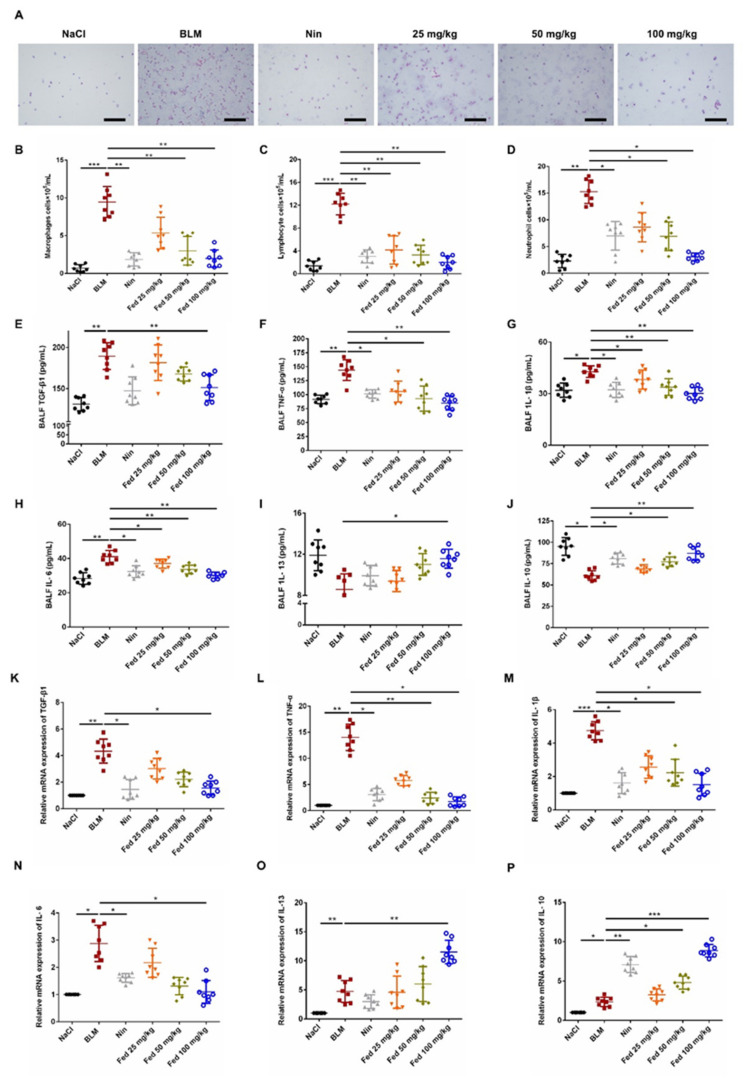
Influence of fedratinib on bleomycin-induced lung inflammation. (**A**) H&E staining of inflammatory cells in the BALF. Representative visual fields of H&E staining from NaCl group, BLM group, Nin group and Fed 25, 50, 100 mg/kg group, respectively. Cells were mainly macrophages, neutrophils and lymphocytes. Original magnification, ×200. (**B**–**D**) Macrophages, neutrophils and lymphocytes from BALF in each group were counted on day 14. (**E**–**J**) The expression of inflammatory factors including TGF-β1, TNF-α, IL-1β, IL-6, IL-13 and IL-10 in BALF were detected using an ELISA kit. (**K**–**P**) mRNA expression of TGF-β1, TNF-α, IL-1β, IL-6, IL-13 and IL-10 in the lung tissues was analyzed by real-time PCR. Data are presented as the means ± SD (*n* = 8). Comparisons between groups were achieved using one-way analysis of variance (ANOVA). * *p* < 0.05, ** *p* < 0.01, *** *p* < 0.001.

Persistence of inflammatory responses accelerates fibrosis progression. Therefore, we challenged mice with BLM to evaluate the anti-inflammation effects of Fed. H&E staining showed that the number of inflammatory cells in the Fed-treated group was remarkably decreased compared to that in the BLM-only group (Figure 7A). The inflammatory cell counts revealed a similar trend, and the Fed high-dose group had a significantly lower number of macrophages (~70% lesser), lymphocytes (~84% lesser) and neutrophils (~86% lesser) (Figure 7B–D). We also measured the cytokine secretion using an ELISA assay. As shown in Figure 6, in the bronchoalveolar lavage fluid (BALF), the profibrotic factors TGF-β1, TNF-α, IL-1β and IL-6 were significantly decreased, while antifibrotic factors IL-10 and IL-13 were elevated (Figure 7E–J). Similar trends of mRNA levels were observed in homogenized lung tissue (Figure 7K–P). These data suggest that Fed can alleviate BLM-induced pulmonary inflammation.

### 2.5. Fed Alleviates Myofibroblast Activation in Bleomycin-Induced Mice by Inhibiting Both JAK2/STAT3 and TGF-β1 Signaling

**Figure 8:**
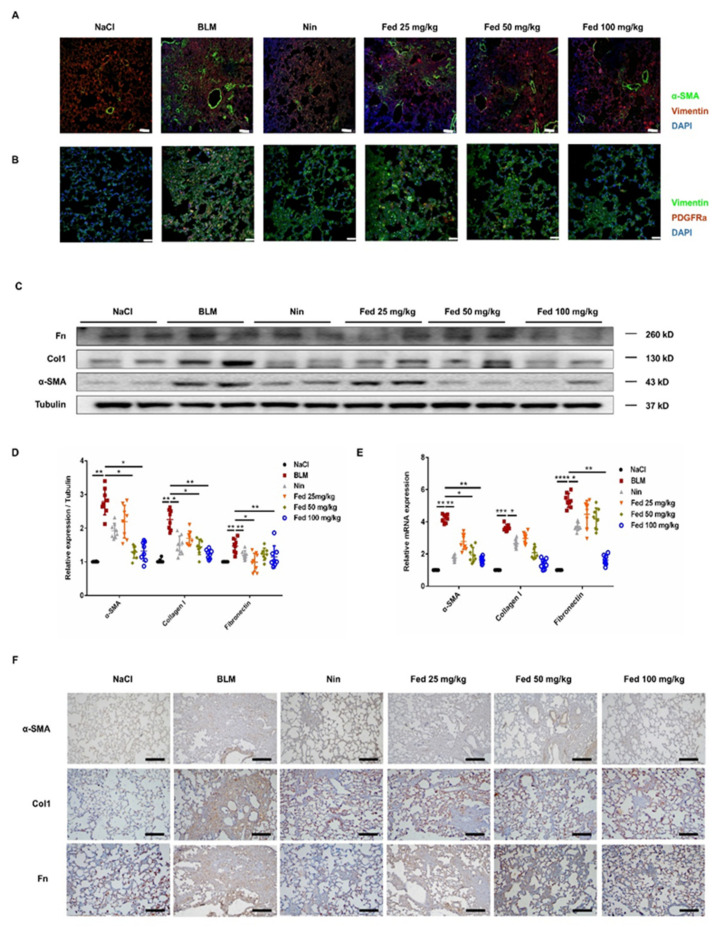
Fedratinib alleviates myofibroblast activation in bleomycin-induced mice through inhibiting both JAK2/STAT3 and TGF-β1 signaling. (**A**) Immunofluorescence staining of α-SMA and vimentin in the lung tissues. (**B**) Immunofluorescence staining of PDGFRα and vimentin in the lung tissues. (**C**,**D**) Protein levels of α-SMA,Col1 and Fn in the lung tissues. (**E**) mRNA expression of α-SMA, Col1 and Fn in the lung tissues. (**F**) Immunohistochemical staining of α-SMA, Col1 and Fn in the lung tissues. Scale bar = 50 μM. Data are presented as the means ± SD (*n* = 8). Comparisons between groups were achieved using one-way analysis of variance (ANOVA). * *p* < 0.05, ** *p* < 0.01, *** *p* < 0.001, **** *p* < 0.0001.

To examine whether Fed attenuates the fibrotic phenotype by decreasing myofibroblast accumulation and activation, we first performed the immunofluorescence on lung sections and labeled myofibroblasts with their marker, α-SMA, mesenchymal cells with their marker, vimentin. As shown in Figure 8, the Fed-treated group displayed a decreased myofibroblast accumulation compared with that in the BLM group. Since PDGFRα + fibroblasts also contribute to collagen deposition in the bleomycin-induced pulmonary fibrosis model [21], we also performed double staining of PDGFRα and vimentin, and results showed that Fed treatment decreased the PDGFRα + fibroblasts compared with that in the BLM group. We further analyzed the mRNA and protein levels of myofibroblast activation markers, α-SMA, Col1 and Fn, and the results consistently showed that Fed significantly decreased BLM-induced overexpression of these factors (Figure 8C–E). Finally, we examined the immunohistochemistry of these factors, and the results confirmed our findings above (Figure 8F). These findings suggest that Fed protects against fibrogenesis in vivo by inhibiting myofibroblast accumulation and activation.

**Figure 9:**
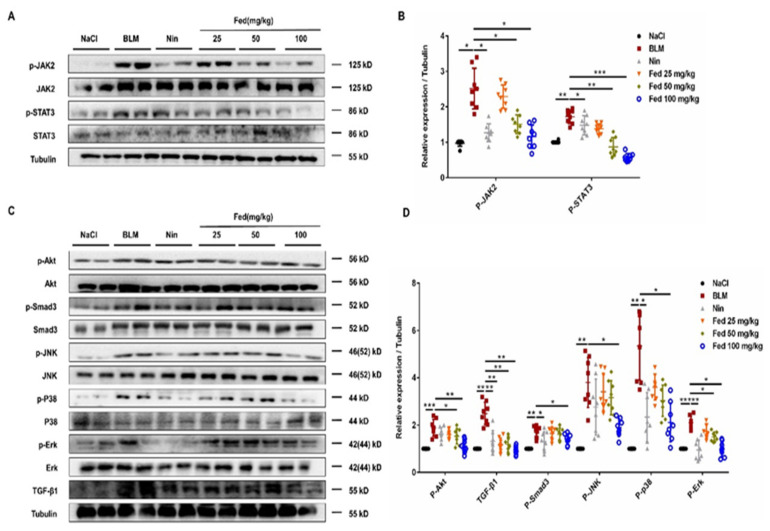
Fedratinib alleviates myofibroblast activation in bleomycin-induced mice by inhibiting both JAK2/STAT3 and TGF-β1 signaling. (**A**) Western blot analysis of the protein levels of JAK2 and STAT3 in lung tissues. (**B**) Densitometric analysis of P-JAK2 and P-STAT3 in the immunoblots, using tubulin as the internal reference. (**C**,**D**) Western blot analysis of the protein levels of Smad3, P38, JNK, Erk, Akt and TGF-β1 in lung tissues. Data are presented as the means ± SD (*n* = 8). Comparisons between groups were achieved using one-way analysis of variance (ANOVA). * *p* < 0.05, ** *p* < 0.01, *** *p* < 0.001.

We further analyzed whether this effect is associated with the suppression of JAK2/STAT3 or TGF-β1/Smad and non-Smad signaling. We first accessed the JAK2/STAT3 activation in lung tissues 14 days after BLM treatment, and the results revealed a significantly decreased phosphorylation level of JAK2 (Fed 100 mg/kg vs. BLM, ~54% lower) and STAT3 (Fed 100 mg/kg vs. BLM, ~78% lower) in the Fed-treated group (Figure 9A,B). This is not a surprise since Fed is a JAK2-selective inhibitor. Meanwhile, we analyzed whether Fed also suppress TGF-β1/Smad and non-Smad signaling in vivo. Phosphorylation levels of Smad3, Akt, Erk, JNK and P38 were lower in the Fed-treated group (Figure 9C,D). These results together suggest that Fed displays antifibrotic effects by inhibiting myofibroblast accumulation and activation, and this fact is associated with the suppression of both JAK2/STAT3 and TGF-β1/Smad and non-Smad signaling.

## 3. Discussion

**Figure 10:**
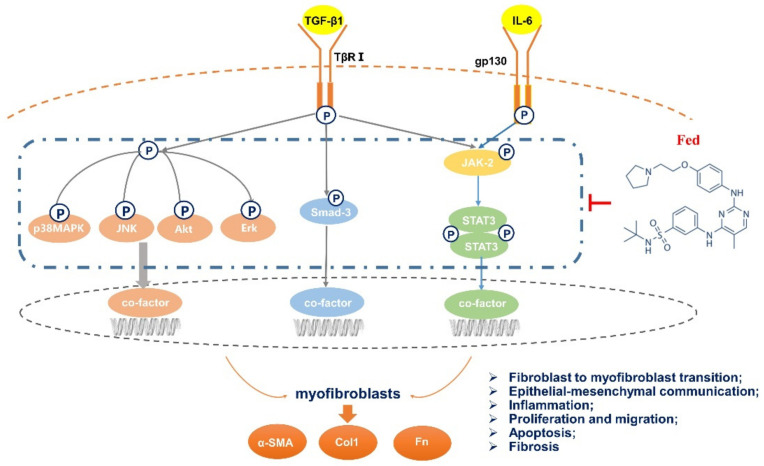
Mechanisms of the anti-pulmonary-fibrosis effect of fedratinib. Fedratinib improves bleomycin-induced pulmonary fibrosis in mice by inhibiting aberrant mesenchymal marker expression of AECs, fibroblast-to-myofibroblast transition, inflammation and apoptosis by targeting the JAK2 receptor and TGF-β1 downstream Smad3 and MAPK signaling pathways.

In this study, we demonstrated the anti-pulmonary-fibrosis and anti-inflammatory effects of Fed through suppressing both JAK2/STAT3 and TGF-β1 signaling. JAK/STAT hyperactivation has been reported to promote fibrosis progression by inducing senescence [22], stimulating NK cell cytotoxicity [23] and impeding autophagic flux [24]. JAK2 is a novel regulator of TGF-β1, and JAK2/STAT3 signaling is widely considered to be necessary in TGF-β1-induced abnormal wound healing of epithelial cells and fibroblast-to-myofibroblast transition. Here, we found that Fed inhibited IL-6- or TGF-β1-induced abnormal wound healing of epithelial cells and fibroblast-to-myofibroblast transition, reduced TGF-β1-induced myofibroblast activation and accumulation and, finally, alleviated bleomycin-induced mice pulmonary fibrosis and inflammation. These data suggest a role for Fed in treating fibrotic diseases (Figure 10).

The JAK2/STAT3 signaling pathway has a prominent role in mediating many of the effects of IL-6 on cell proliferation, invasion and chronic inflammation [25,26]. Elevated levels of serum IL-6 are seen in patients with fibrosis diseases [27,28], and targeting IL-6/STAT3 signaling is beneficial for fibrotic disease [29,30] treatment. We found that IL-6-induced JAK2/STAT3 activation contributed to mesenchymal marker expression in epithelial cells and myofibroblast transition in fibroblasts, which is in line with the previous results [11,31]. It is not a surprise that Fed exerts such effects since it is a selective JAK2 tyrosine kinase inhibitor. Notably, IL-6-induced activation of STAT3 can further mediate the production of cytokines such as IL-6, TGF-β1 and IL-1β [32], thus forming a vicious circle to promote fibrogenesis. Fed regulates the secretion of these cytokines, thus transforming the vicious circle to a virtuous one and preventing the epithelial cells from further injury. Our findings are in line with a previous study that Fed inhibits LPS-induced inflammatory cytokine IL-6, IL-1β and TNF-α secretion in macrophages [33], together suggesting the anti-inflammation effect of Fed.

Activation of TGF-β1 has been shown to play a central role in regulating tissue fibrosis. TGF-β1-induced myofibroblast activation and apoptosis-resistance phenotype contributes to ECM deposition and fibrosis foci formation [34]. Here, we found that Fed suppressed TGF-β1-induced myofibroblast migration, activation and apoptosis resistance by decreasing the phosphorylation levels of Smad3, Akt, Erk, JNK and P38. It is still unclear whether Fed displays such effects by interfering with the crosstalk between JAK2/STAT3 and TGF-β1 signaling or by directly targeting the upstream of TGF-β1 signaling. TGF-β1 may activate STAT3 indirectly by inducing IL-6 or other cytokines [35]. Thus, inhibition of JAK2 by Fed will consequently decrease the activation of TGF-β1 signaling. However, although Fed is a selective tyrosine kinase inhibitor of JAK2, it binds to the ATP binding sites of JAK2 [14], which share a similar structural model with most of the kinase receptors. This makes it possible for Fed to exert inhibitory effects on other kinase receptors. Recent evidence also indicates that in fibroblasts from IPF patients, TGF-β1 activates STAT3 via a Smad2/3-dependent mechanism and independent of JAK2 [11]. This fact also indicates the possibility that the inhibitory effect of Fed on TGF-β1 signaling might be independent of JAK2.

Fibrotic diseases often begin with an insult to the epithelium and initiate chronic inflammation [36]. In response to the injury, released chemokines promote the infiltration of immune cells and release profibrotic cytokines [37]. These cytokines then activate fibroblasts to become myofibroblasts that in turn secrete collagen and other ECM proteins that stiffen the lung. Blocking JAK2/STAT3 signaling has been demonstrated to reduce both inflammation and fibrosis in murine model [12,33]. Here, we found that Fed reduced bleomycin-induced inflammatory response, decreased infiltration of immune cells and downregulated the secretion of profibrotic cytokines such as TGF-β1, TNF-α, IL-1β and IL-6. Meanwhile, Fed alleviated bleomycin-induced mice pulmonary fibrosis, manifesting as a decrease in extracellular matrix deposition, reduction in hydroxyproline biosynthesis and restoration in lung functions. Decreased chemokine secretion prevents the epithelium from further injury, thus alleviating myofibroblast activation which together confirms the antifibrosis effect of Fed.

Although we only examined the effect of Fed in a murine model, we believe it to be also beneficial in treating patients with pulmonary fibrosis. Since Fed has already been approved for treating myelofibrosis, its safety is well promised [38,39]. Meanwhile, the significant antimyelofibrosis effects [40] also indicate the potential effect of Fed in treating fibrosis in other types of organs. Our study indicates a potential clinical value of Fed for pulmonary fibrosis treatment.

## 4. Materials and Methods

### 4.1. Antibodies and Reagents

Fed was purchased from TargetMol, and the purity of Fed was 99%. For each cell experiment, Fed was freshly prepared by dissolving in DMSO (DAMAO CHEMICAL). Bleomycin (BLM) was acquired from Nippon Kayaku (Tokyo, Japan). TRIzol reagent was from Ambion Life Technology. DEPC-treated H_2_O and SYBR Green Real-time PCR Master Mix were from Life Technologies. M-MLV reverse transcriptase was purchased from Promega, and PCR Buffer without MgCl_2_ and MgCl_2_ stock solution were acquired from Roche. In addition, recombinant ribonuclease inhibitor, dATP, dTTP, dCTP and dGTP were acquired from Takara. RIPA lysis buffer (middle) and the BCA kit were purchased from Beyotime Biotechnology. The primary antibodies described in the study include the following: anti-E-cadherin, anti-vimentin, anti-N-cadherin, anti-fibronectin and anti-collagen I (Affinity Biosciences, OH, USA) and GAPDH, Smad3, p-Smad3, Erk, p-Erk, JNK, p-JNK, P38, p-P38, p-Akt and Akt antibody (Cell Signaling Technology, Boston, MA, USA); α-SMA, p-STAT3, STAT3, p-JAK2, JAK2, Bax, Bcl-2, cleaved caspase-3 and caspase-3 antibody were from Santa Cruz Biotechnology (Shanghai, China) and Protein-Tech (Shanghai, China), respectively. PDGFRα antibody was from Bioss (Beijing, China). The secondary antibodies, antirabbit IgG (H + L) and antimouse IgG (H + L), were from Applygen (Beijing, China). ELISA kits were purchased from Jianglaibio (Shanghai, China). The apoptosis kit was purchased from Solarbio (Beijing, China).

### 4.2. Cell Culture

Mouse fibroblast cells (NIH3T3, purchased from ATCC) were grown in DMEM (KeyGEN Biotech, Nanjing, China) supplemented with 10% fetal bovine serum (FBS, ExCell Bio, Shanghai, China). MLE-12 cells maintained in DMEM/F-12 (Gibco, Beijing, China) with 10% FBS. Cells were maintained at 37 °C with 5% CO_2_ in a humidified atmosphere.

### 4.3. Animals

C57BL/6 mice at 7–8 weeks were obtained from Vital River Laboratory Animal Technology Co., Ltd. (Beijing, China). Mice were housed in a room at a temperature of 22 to 26 °C and a humidity between 40% and 70%, with a 12 h light/dark cycle. Mice were free to eat and drink. All animal care and experimental procedures complied with guidelines approved by the Institutional Animal Care and Use Committee (IACUC) of Nankai University (permit no. SYXK 2014-0003). Animal studies are reported in compliance with the ARRIVE guidelines [41,42].

### 4.4. Bleomycin Administration

The model was constructed according to the classic pulmonary fibrosis model of mice [43]. First, the mice were anesthetized by intraperitoneal injection and weighed. Then the mouse belt was fixed to the operating table, the neck of the mouse was disinfected with 75% alcohol, a wound of about 1 cm was cut with a scalpel and the muscle tissue was carefully stripped to expose its trachea clearly. At a dose of 2 U/kg, we used a sterile insulin syringe (with about 10 units of air column) to suck the corresponding volume of the prepared BLM solution and slowly and parallelly injected the BLM into the mouse trachea from the cartilage ring. We put the mouse body into an upright position immediately and beat the back of the mouse several times (in order to distribute the BLM evenly in the lung tissue). The 60 mice were divided into six groups, with 10 mice per group randomly: control group, BLM group, positive control group (BLM + nintedanib, 100 mg/kg), low-dose Fed (BLM + Fed 25 mg/kg), medium-dose Fed (BLM + Fed, 50 mg/kg) and high-dose Fed (BLM + Fed, 100 mg/kg). Starting from 7 days after BLM injury, Fed was given daily by intragastric administration for 1 week. The positive control was nintedanib. The control group and the model group were given the same volume of control substance (5% CMC-Na) using the same administration regimen and route. On the 14th day after bleomycin administration, mice were sacrificed to evaluate pulmonary fibrosis and other indicators.

### 4.5. Pulmonary Function Testing

The mice were anesthetized by intraperitoneal injection of 10% chloral hydrate according to the body weight of the mice, and the trachea was fixed and exposed, then a Y-type tracheal cannula (2.5 mm inner diameter) was inserted for intubation. After the surgery, the mice were placed in a supine position with their whole body inside the plethysmographic chamber to analyze pulmonary function using the AniRes 2005 system (Beijing Biolab, Beijing, China). One branch of the Y-type tracheal cannula was connected to a venthole on the chamber wall, and the venthole was connected to a ventilator from the outside of the chamber by polyethylene tubing. Natural air entered the animal’s body through a ventilator, 80 breaths per minute. Another branch of the intubation was connected to the pressure detection channel of the AniRes 2005. The peak respiratory pressure was maintained at 10 to 16 cm of water. By detecting the pressure associated with ventilation, the cavity was replaced by an AniRes 2005 automatic system. The display pulmonary function parameters such as forced vital capacity (FVC), dynamic compliance (Cdyn), inspiratory resistance (Ri), and expiratory resistance (Re) were then calculated.

### 4.6. Hydroxyproline Measurement

The three largest lobes of the right lung of the mouse were placed in a 5 mL ampoule, dried in the oven, hydrolyzed with hydrochloric acid, adjusted to pH 6.5−8.0 with sodium hydroxide and volume was fixed to 10 mL with 1× PBS [44]. Each tube was comprised of 350 μL H_2_O, 50 μL sample, and 200 μL Chloramine-T. The samples were vortexed and incubated at room temperature for 20 min. Then, 200 µL of perchloric acid was added, and the samples were vortexed and incubated at room temperature for 5 min. P-DMAB (200 μL) was added, and the samples were vortexed and incubated at 60 °C in a water bath for 20 min. The samples were cooled, 200 μL of each sample was transferred to 96-well plates in triplicate, and the absorbance was measured at 577 nm.

### 4.7. Histological Examination

The left lung of the mice was fixed in formalin for 48 h, then dehydrated, embedded and sectioned. Then, lung sections (4 μm) were prepared and stained with hematoxylin–eosin (H&E) and Masson’s trichrome. Quantification of pulmonary fibrosis was performed as described previously [45]. In brief, images were photographed with an upright transmission fluorescence microscope (Olympus) and analyzed by Image-Pro Plus Version 6.0 (Media Cybernetics, Inc, American). The software selection tool can select the entire lung tissue area and automatically calculate the total pixels, P_w_, of the region. It can then use the same method to calculate the total pixels, P_f_, of the fibrosis region (fibrosis ratio = fibrosis area total pixel, P_f_/total lung total pixel, P_w_).

### 4.8. Cell Viability Analysis

Cell viability was determined using 3-(4,5-dimethylthiazol-2-yl)-2,5-diphenyltetrazolium bromide (MTT). NIH-3T3 cells were seeded in 96-well plates and exposed to Fed (0 to10 µM) for 24 h. Then, 10 µL of MTT (5 mg/mL, China) was added to the NIH-3T3 cells for 4 h, and 120 µL DMSO was loaded into per well. The absorbance value was determined at 570 nm.

### 4.9. Wound Healing Assay

According to reference [46], NIH3T3 cells were spread in 6-well plates. After 24 h, serum starvation was performed. The wounds were treated with a 200 μL sterile spear head and marked on the back of the culture dish. Then NIH3T3 cells were cultured with Fed (0.5, 1, 2 µM) and TGF-β1 (5 ng/mL). The scratch was observed at 0, 12, 24 and 48 h using an inverted optical microscope.

### 4.10. Quantitative Real-Time PCR (qRT-PCR)

Total RNA was extracted from NIH3T3 cells, MLE12 cells and tissues using TRIzol reagent, and RNA was isolated [47]. We obtained cDNA from total RNA by reverse transcription, qRT-PCR was performed using SYBR GreenER qPCR SuperMix Universal (Invitrogen) according to the manufacturer’s protocols. The relative quantification of gene expression was measured relative to the endogenous reference gene (mouse: *GAPDH*) using the comparative CT method. The primers optimized for real-time PCR assays are listed in Table 1.

### 4.11. Western Blotting Analysis

Proteins were extracted from cells or lung tissue according to standard protocols, as described earlier [48]. All of the protein was extracted from lung tissue homogenates or cells using radio-immunoprecipitation assay (RIPA) lysis buffer containing phenylmethylsulfonyl fluoride (PMSF) and sodium fluoride (NaF). After electrophoresis and membrane transfer, the immunoblots were probed with the following primary antibodies: GAPDH, tubulin, α-SMA, collagen I, fibronectin, E-cadherin, vimentin, N-cadherin, p-Smad3, Smad3, p-Erk, Erk, p-Akt, Akt, p-P38 MAPK, p38 MAPK, p-JNK, JNK, p-STAT3, STAT3, p-JAK2, JAK2, Bax, Bcl-2, cleaved caspase-3 and caspase-3. The secondary antibodies were goat antirabbit or goat antimouse horseradish-peroxidase-conjugated antibodies. Enhanced chemiluminescence reagent was used for detection, and blots were scanned using AlphaView SA software.

### 4.12. Immunofluorescence Staining

The paraffin-embedded lung tissue was sectioned, dried and dewaxed. The slices were put into the antigenic repair solution (0.01 M citrate buffer) and boiled for 2 min. After this, cells were permeabilized with 0.2% Triton X-100 (Fluorochem, Hadfield, UK) for 30 min and blocked with 5% BSA in phosphate-buffered saline (TBS-T) for 60 min in a humidified chamber. Lung sections were incubated with α-SMA (1:200 dilution) and vimentin mixed antibody (1:200 dilution), PDGFRα (1:200 dilution) and vimentin mixed antibody (1:200 dilution), respectively, and incubated overnight at 4 °C. After washing with 1× PBS-T, lung slices were incubated with TRITC-conjugated or FITC-conjugated secondary antibody. Lung slices were counterstained with DAPI (Beyotime Biotechnology, Nanjing, China). Phase contrast and fluorescent microscopy was performed using an Olympus IX81 inverted research microscope.

### 4.13. Immunohistochemistry Staining

The paraffin-embedded lung tissue was sectioned, dried and dewaxed. The slices were put into the antigenic repair solution and boiled for 2 min. After washing with PBS, the primary antibody was incubated at 4 °C overnight with an immunohistochemical colorimetric kit. The primary antibodies were as follows: mouse anti-α-SMA antibody (1:100 dilution), rabbit anticollagen I antibody (1:100 dilution) and rabbit antifibronectin (1:100 dilution). After washing three times with PBS, tissue sections were incubated with a DAB Immunohistochemistry Color Development Kit. The lung tissue sections were stained with hematoxylin, and the histological changes and target gene expression of mouse lung tissues were observed under a light microscope.

### 4.14. Bronchoalveolar Lavage

Mice were anesthetized and immobilized with 10% chloral hydrate, their tracheas were separated and lavaged through a blunt needle attached to a syringe that served as a tracheotube in the airway. Next, 1 mL of bronchoalveolar lavage fluid (BALF) was collected and rinsed three times with PBS. The collected BALF was centrifuged at 3000 r/min× *g* for 10 min. After centrifugation, the supernatant was cryopreserved at −80 °C for cytokine analysis. Cell precipitation was lysed with red blood cell lysate, resuspend for 10 min, and then centrifuged at 3000 r/min× *g* for 10 min. Then the lower cells were resuspend with 200 μL 1× PBS. Total cell count was performed using a Countstar automatic cell counting instrument. Macrophages, neutrophils and lymphocytes were counted under an optical microscope, and a 50 μL suspension was smeared and stained with H&E staining.

### 4.15. ELISA Detection

MLE12 cells were treated with BLM (5 U) and Fed (0.5, 1 and 2 μM) for 24 h and the cell supernatant was centrifuged at 1000 r× *g* for 20 min for ELISA detection. The mice alveolar lavage fluid was tested using ELISA. The concentration of inflammatory factors including IL-1β, TGF-β1, IL-10, IL-6, TNF-α and IL-13 were detected using ELISA kits (Jianglaibio, Shanghai, China) following the manufacturer’s protocol.

### 4.16. Apoptosis Analysis

The percentage of apoptotic cells was measured by flow cytometry. The cells in the control group, the TGF-β1 stimulation group, and the TGF-β1 (5 ng/mL) + Fed (2 μM) stimulation group were digested with trypsin, and PBS was clear. The cells were resuspend and counted with binding buffer. The apoptotic cells were double stained with FITC and PI and measured by flow cytometry within 1 h.

### 4.17. Data and Statistical Analysis

All statistical analyses were performed using GraphPad prism 7.0 software as the means ± SD (GraphPad Software, Inc., La Jolla, CA, USA). The comparisons were done by one-way analysis of variance (ANOVA) followed by the Tukey–Kramer test to identify significant differences between groups. *p* < 0.05 was considered to be statistically significant.

## Figures and Tables

**Table 1:** Primers sequences for real-time PCR.

Gene	Forward Primer Sequence (5′–3′)	Reverse Primer Sequence (3′–5′)
Mouse *GAPDH*	AGGTCGGTGTGAACGGATTTG	GGGGTCGTTGATGGCAACA
Mouse *α-SMA*	GTCCCAGACATCAGGGAGTAA	GTCCCAGACATCAGGGAGTAA
Mouse *Fibronectin*	TCGGATACTTCAGCGTCAGGA	TCGGATACTTCAGCGTCAGGA
Mouse *Collagen I*	ATGTGGACCCCTCCTGATAGT	ATGTGGACCCCTCCTGATAGT
Mouse *IL-10*	GCTCTTACTGACTGGCATGAG	CGCAGCTCTAGGAGCATGTG
Mouse *IL-6*	TAGTCCTTCCTACCCCAATTTCC	TTGGTCCTTAGCCACTCCTTC
Mouse *IL-1β*	GCAACTGTTCCTGAACTCAACT	ATCTTTTGGGGTCCGTCAACT
Mouse *TNF-α*	CCCTCACACTCAGATCATCTTCT	GCTACGACGTGGGCTACAG
Mouse *IL-13*	CCTGGCTCTTGCTTGCCTT	GGTCTTGTGTGATGTTGCTCA
Mouse *TGF-β1*	CTCCCGTGGCTTCTAGTGC	GCCTTAGTTTGGACAGGATCTG

## Data Availability

The data presented in this study are available on request from the corresponding author.

## Sample Availability

A sample of the compound fedratinib is available from the authors.
